# Unveiling Clinically Significant Anti-Lewis Antibodies: A Case Report

**DOI:** 10.7759/cureus.75975

**Published:** 2024-12-18

**Authors:** Muhammad 'Aqil Nazahah Mohamad Mustafa, Mohammad Faqis Abdul Aziz, Adibah Daud, Sharifah Sakinah Syed Abdul Rahman, Mohammad Hudzaifah Nordin, Razan Hayati Zulkeflee, Sumaiyah Adzahar

**Affiliations:** 1 Department of Pathology and Medical Laboratory, Hospital Sultan Zainal Abidin, Kuala Terengganu, MYS; 2 Department of Pathology and Medical Laboratory, Faculty of Medicine, Universiti Sultan Zainal Abidin, Kuala Terengganu, MYS; 3 Department of Ophthalmology, Faculty of Medicine, Universiti Sultan Zainal Abidin, Kuala Terengganu, MYS; 4 Department of Hematology, School of Medical Sciences, Universiti Sains Malaysia, Kubang Kerian, MYS

**Keywords:** anti-lewis antibodies, blood group, clinical significance antibody, hemolytic transfusion reaction, phenotyping

## Abstract

Lewis antibodies, such as anti-Le^a^ and anti-Le^b^, are commonly encountered in routine immunohematology. They are typically IgM in nature and are generally considered clinically insignificant, as they rarely cause hemolytic transfusion reactions (HTRs) or hemolytic disease of the fetus and newborn (HDFN). However, rare cases have been reported where anti-Lewis antibodies caused mild transfusion reactions. In this case report, we describe a 69-year-old male with sepsis secondary to a neck carbuncle who was found to have clinically significant anti-Lewis antibodies. These antibodies presented a challenge during crossmatching, as only two out of seven units of packed red blood cells were compatible. This case underscores the importance of thorough pre-transfusion testing to ensure safe and effective blood transfusion practices.

## Introduction

The Lewis blood group system comprises antigens found on red blood cells (RBCs) and in body secretions. Antibodies against Lewis antigens are generally considered clinically insignificant, as they typically react at low temperatures and are not associated with hemolytic transfusion reactions (HTRs) or hemolytic disease of the newborn. There are several reasons why the presence of anti-Lewis antibodies is generally clinically insignificant. Firstly, these antibodies are typically immunoglobulin M (IgM) antibodies, and their occurrence when reacting at 37°C is relatively low [1]. Secondly, Lewis antigens are produced under the control of the Se gene and Le gene, involving the type 1 precursor chain rather than type 2 [1]. These antigens are absorbed from the plasma and passively attached to the red cell membrane. Consequently, anti-Lewis antibodies bind to the Lewis antigen instead of directly binding to the red cell membrane [1]. However, although exceedingly rare, clinically significant anti-Lewis antibodies have been reported in the literature and can present challenges in blood transfusion management.

## Case presentation

**Figure 1:**
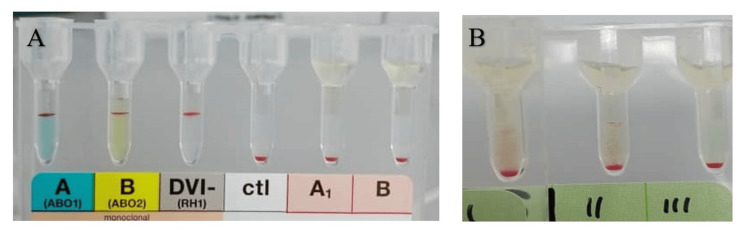
(A) Patient blood group was AB, rhesus positive; (B) Antibody screening was positive in cells I and II.

A 69-year-old male was admitted to our hospital due to sepsis secondary to a neck carbuncle and planned for saucerization of the carbuncle. Pre-transfusion testing showed his blood group was AB, rhesus positive (Figure 1A). However, the antibody screening revealed positive results for cells I and II, indicating the presence of an antibody (Figure 1B). His blood sample was sent to the reference laboratory for antibody identification. The results revealed that the alloantibodies in his plasma were identified as clinically significant anti-Le^a^ and anti-Le^b^, reactive at 37°C, posing a significant challenge in selecting compatible blood for transfusion. Crossmatching with seven group AB, rhesus-positive packed red cells, two units were found to be compatible and were reserved for the patient. The patient's RBCs were typed as Le^a^ and Le^b^ negative. All the investigations are summarized in Table 1. The patient underwent successful saucerization of the neck carbuncle with minimal intraoperative bleeding. Fortunately, no blood transfusion was required during or after the surgery, as the patient's hemoglobin levels remained stable.

**Table 1:** A tabulated overview of the immunohematological investigations conducted for the patient during hospital admission.

Immunohematology investigations	Results
ABO typing (gel card method)	AB
Rh(D) (gel card method)	Positive
Antibody screening	Positive for cell I and cell II
Antibody Identification	Anti‑Le^a^ and anti‑Le^b^
Lewis phenotype	Le(a–b–)
Direct Coombs test	Negative
Crossmatch compatible	2 out of 7 units of red blood cell

## Discussion

The Lewis blood group system is a classification system for human blood based on the presence or absence of specific antigens on the surface of RBCs. Unlike other blood group systems, the Lewis system antigens are not inherent to RBCs but are derived from substances found in body fluids, mainly plasma. These substances, known as Lewis antigens, are soluble glycoproteins that are adsorbed onto the surface of RBCs from the plasma [2]. The Lewis blood group system comprises six antigens: Le^a^, Le^b^, Le^ab^, Le^bH^, ALe^b^, and BLe^b^. Among these, the Le^a^ and Le^b^ antigens hold particular significance and are commonly encountered in clinical settings. Consequently, four distinct Lewis antigen phenotypes have been identified: Le(a-b-), Le(a-b+), Le(a+b-), and Le(a+b+) [2,3].

Lewis antibodies are often naturally occurring, made by Le(a-b-) persons that occur without known RBC stimulus. They are generally IgM and do not cross the placenta. Anti-Le^a^ and anti-Le^b^ antibodies may coexist and are commonly found in the plasma of pregnant women who transiently display the Le(a-b-) phenotype due to hemodynamic alterations in pregnancy [4]. These antibodies can be neutralized by Lewis substances present in saliva or plasma [5].

In general, clinically significant antibodies are those that react at 37°C in vitro and during the indirect antiglobulin test (IAT) phase, typically belonging to the IgG class. However, antibodies related to the Lewis blood group system are often regarded as naturally occurring and primarily belonging to the IgM class fraction [6]. These antibodies typically react at temperatures below 37°C and are not considered clinically significant [7]. Regardless of the Lewis phenotype, RBCs compatible at 37°C are expected to have normal in vivo survival. Therefore, transfusing antigen-negative RBCs for patients with antibodies against Lewis antigens is not typically considered necessary.

Anti-Le^a^ is the most encountered among the Lewis antibodies and sometimes reacts at 37°C and in the IAT. Severe cases of HTRs have been documented in patients with anti-Le^a^ who received transfusions of Le(a+) RBCs. These reactions exhibit rapid onset following transfusion and present with a spectrum of symptoms indicative of intravascular RBC destruction mediated by complement [8,9]. These hemolysins are primarily of the IgM type and may activate complement leading to in vivo or in vitro hemolysis [10]. For such cases, providing Le^a ^antigen-negative RBC units crossmatched at 37°C may be favorable to prevent further HTR. In contrast, anti-Le^b^ is not as potent as anti-Le^a^ and typically manifests as an IgM agglutinin that can bind complement.

We present our experience with a male patient who was discovered to have clinically significant Lewis antibodies despite no prior blood transfusions. This is uncommon as most Lewis antibodies are deemed clinically insignificant. However, the patient's Lewis antibodies exhibited reactivity at both room temperature and at 37°C, indicating an IgM antibody with a broad thermal amplitude. This characteristic raises concerns for potential acute intravascular hemolysis under physiological conditions [1,5]. In this case, no further test was conducted to determine whether the reactive antibody was of the IgG or IgM class as the test did not change the patient's blood management. The incidence of the Le(a-b-) phenotype in the Malaysian population is approximately 22.0% in Malays, 12.5% in Chinese, and 24.2% in Indians [11]. Therefore, finding Lewis antigen-negative RBC units may be challenging since most blood donors express Lewis antigens.

## Conclusions

Patients with clinically significant Lewis antibodies should receive compatible RBC units that are negative for the Lewis antigen to ensure patient safety while maximizing therapeutic transfusion benefits.

## References

[1] Marrero GT, Rose WN (2023). Warm-reacting anti-Lewis a antibody: a case report. Med Res Publ.

[2] Subramaniyan R (2023). Serological characteristics of Lewis antibodies and their clinical significance - a case series. Hematol Transfus Cell Ther.

[3] Wei X, Liu F, Ran D, Yin P, Qu L (2023). Situation analysis and blood transfusion strategy of Lewis antibodies in Hunan Province. Allergol Immunopathol (Madr).

[4] Gayathri AM, Gupta D (2020). Case series investigation on the Lewis system antibodies encountered during a routine screening in a tertiary care hospital-based blood center. Asian J Transfus Sci.

[5] Harmening DM (2019). Modern Blood Banking & Transfusion Practices. Modern Blood Banking & Transfusion Practices..

[6] Makroo RN, Arora B, Bhatia A, Chowdhry M, Luka RN (2014). Clinical significance of antibody specificities to M, N and Lewis blood group system. Asian J Transfus Sci.

[7] Negi G, Malhotra S, Meinia SK, Kaur D, Rai D (2020). Adding further evidence for clinically significant anti-Le(b) antibody in a voluntary blood donor. Asian J Transfus Sci.

[8] Höglund P, Rosengren-Lindquist R, Wikman AT (2013). A severe haemolytic transfusion reaction caused by anti-Le(a) active at 37 °C. Blood Transfus.

[9] Irani MS, Figueroa D, Savage G (2015). Acute hemolytic transfusion reaction due to anti-Le(b). Transfusion.

[10] Das S, Chakrabarty R (2018). Optimization of blood safety through essential characterization of naturally occurring Lewis antibody. Glob J Transfus Med.

[11] Musa RH, Ahmed SA, Hashim H, Ayob Y, Asidin NH, Choo PY, Al-Joudi FS (2012). Red cell phenotyping of blood from donors at the National blood center of Malaysia. Asian J Transfus Sci.

